# H^2^GnnDTI: hierarchical heterogeneous graph neural networks for drug–target interaction prediction

**DOI:** 10.1093/bioinformatics/btaf117

**Published:** 2025-03-17

**Authors:** Yueying Jing, Dongxue Zhang, Limin Li

**Affiliations:** School of Mathematics and Statistics, Xi’an Jiaotong University, Xi'an, Shaanxi 710049, China; School of Mathematics and Statistics, Xi’an Jiaotong University, Xi'an, Shaanxi 710049, China; School of Mathematics and Statistics, Xi’an Jiaotong University, Xi'an, Shaanxi 710049, China

## Abstract

**Motivation:**

Identifying drug–target interactions (DTIs) is a crucial step in drug repurposing and drug discovery. The significant increase in demand and the expensive nature for experimentally identifying DTIs necessitate computational tools for automated prediction and comprehension of DTIs. Despite recent advancements, current methods fail to fully leverage the hierarchical information in DTIs.

**Results:**

Here, we introduce H^2^GnnDTI, a novel two-level hierarchical heterogeneous graph learning model to predict DTIs, by integrating the structures of drugs and proteins via a low-level view GNN and a high-level view GNN. The hierarchical graph consists of high-level heterogeneous nodes representing drugs and proteins, connected by edges representing known DTIs. Each drug or protein node is further detailed in a low-level graph, where nodes represent molecules within each drug or amino acids within each protein, accompanied by their respective chemical descriptors. Two distinct low-level graph neural networks are first deployed to capture structural and chemical features specific to drugs and proteins from these low-level graphs. Subsequently, a high-level graph encoder (GE) is used to comprehensively capture and merge interactive features pertaining to drugs and proteins from the high-level graph. The high-level encoder incorporates a structure and attribute information fusion module designed to explicitly integrate representations acquired from both a feature encoder and a GE, facilitating consensus representation learning. Extensive experiments conducted on three benchmark datasets have shown that our proposed H^2^GnnDTI model consistently outperforms state-of-the-art deep learning methods.

**Availability and implementation:**

The codes are freely available at https://github.com/LiminLi-xjtu/H2GnnDTI.

## 1 Introduction

The identification of drug–target interactions (DTIs) is an important part of pharmacology and drug discovery. The traditional process of drug development is time-consuming and costly, whereas very few drugs ever make it to the clinic (Zhao *et al.* 2021). With the production of a large amount of biological activity data in recent years, predicting DTIs through deep learning technology has become a popular research topic. Many studies have adopted computational methods to effectively identify drug target associations on a large scale (Tsubaki *et al.* 2019, Wang *et al.* 2020). Recently, deep learning-based methods have shown good performance, but there are still two challenges: how to clearly model and learn local representations between drugs and targets for better prediction, and how to optimize the generalization performance of novel (unseen) proteins and drugs.

Over the past decades, many end-to-end models have been applied to study DTIs. DeepConv-DTI (Lee *et al.* 2019) utilizes multi-scale one-dimensional CNN (1D-CNN) layers to obtain protein features and get Extended Connectivity Fingerprints (ECFP) (Rogers and Hahn 2010) of drugs as input. To capture any relationship among atoms in a sequence, a Transformer-based DTI model (Kalakoti *et al.* 2022) uses multi-layered bidirectional Transformer encoders (Vaswani *et al.* 2017) to learn the high-dimensional structure of a molecule from the simplified molecular-input line entry system (SMILES) string. In recent years, graph neural networks (GNNs) have achieved good performance in node/graph representation learning by simultaneously utilizing node attributes and graph high-levelology. For instance, Gu *et al.* (2022) proposed a heterogeneous GNN approach to integrate large-scale heterogeneous biological networks in the field of drug repositioning, which substantially improves prediction accuracy by deeply mining relationships between diverse biological entities. The DrugBAN (Bai *et al.* 2023) works on drug molecular graphs and target protein sequences to perform prediction, and proposes an interpretable bilinear attention network with domain adaptation to improve drug–target prediction accuracy.

While previous efforts have shown promising results by utilizing both types of information, these methods typically focus on a single view of either sequences or graphs. Few approaches effectively model the natural hierarchy of DTIs by integrating and connecting both views. Furthermore, existing methods often lack a suitable mechanism for information fusion of drug molecule graphs and protein residue graphs. Information from multiple sources is commonly aligned or concatenated, which limits the depth of interaction and integration of information.

To address the aforementioned challenges, we propose an end-to-end bio-inspired approach named H^2^GnnDTI for predicting DTIs. The hierarchical graph model has previously been used in a homogeneous manner to investigate protein-protein interactions (Gao *et al.* 2023), demonstrating effective performance in capturing protein features for predicting homogeneous interactions. Howerver, DTI prediction encompasses a more complicated task as it involves predicting heterogeneous interactions between drugs and targets, where the hierarchical knowledge of structural information plays a crucial role in understanding the molecular intricacies of DTIs. H^2^GnnDTI utilizes a hierarchical heterogeneous graph and incorporates both low-level and high-level GNNs (Kipf and Welling 2017, Xu *et al.* 2018) to capture structural and interactive features of drugs and proteins. This approach could address the limitations in previous prediction methods. On the one hand, high-level and low-level GNNs enable information propagation from a molecule in a drug graph or an amino acid in a protein graph to drugs or proteins in the DTI graph, and thus make full use of hierarchical structure to learn efficient representations of drug and protein structures. On the other hand, within the high-level GNN, a structure and attribute information fusion module is used to effectively integrate attribute and structural information derived from both the feature encoder (FE) and the graph encoder (GE), thereby enhancing the construction of comprehensive and accurate representations. Our H^2^GnnDTI’s propagation mechanism facilitates the recovery of network properties, such as node degrees and community partitions, and accurately predicts DTIs.

The contributions of this work are summarized as follows:

1. H^2^GnnDTI utilizes two levels of GNNs to model drug/protein structures and DTI networks. In the low-level view, it represents drug and protein structures using GNNs: atoms as nodes and chemical bonds or amino acid residues as edges in their respective graphs. This approach integrates atom/residue-level properties and 3D structures synergistically through low-level view GNN (LGNN). In the high-level view, H^2^GnnDTI *constructs* the DTI graph where drug and protein graphs serve as nodes and interactions as edges, enabling high-level view GNN (HGNN) to learn drug-protein relationships effectively.
2. Using an end-to-end training approach, H^2^GnnDTI leverages synergies from both perspectives. The low-level view enriches the high-level view by supplying detailed drug/protein representations, enhancing the accuracy of learned interactive features. Simultaneously, insights gained from drug/protein relationships learned in the high-level view inform refinements in the low-level view, thereby improving the establishment of comprehensive drug/protein representations.
3. The structure and attribute information fusion (SAIF) module is used to enhance the elaboration of information from two distinct sources. It integrates sample embeddings from both structural and attribute levels to facilitate consensus representation learning.

We evaluate the performance of our proposed model and compare it with several state-of-the-art models across three datasets. Experimental results consistently demonstrate that H^2^GnnDTI outperforms these models in terms of prediction accuracy, highlighting the strong capability of its hierarchical heterogeneous graph structure in DTI prediction. These findings suggest that our approach holds promise for advancing the understanding of drug repurposing mechanisms.

## 2 Materials and methods

**Figure 1.**
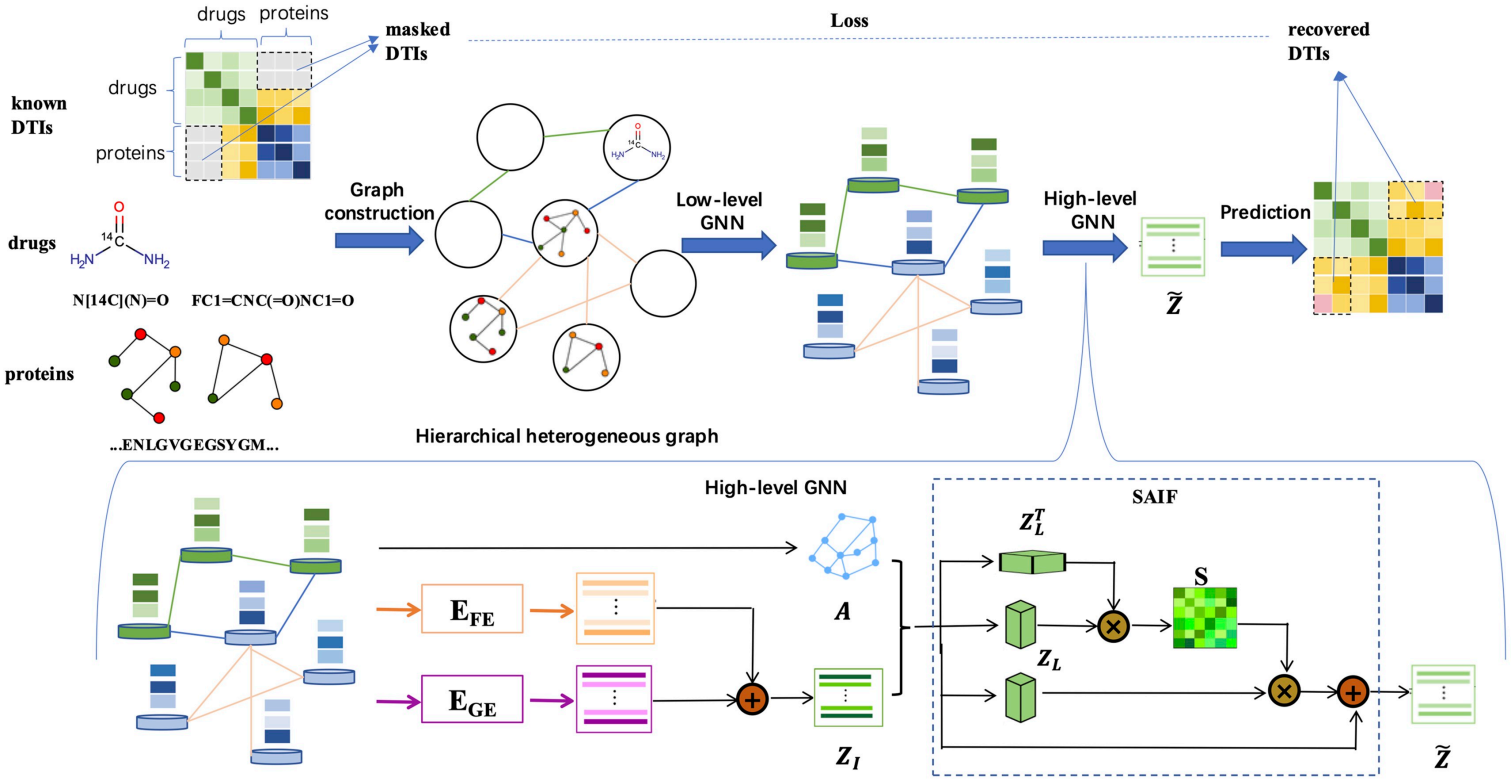
The model structure of H^2^GnnDTI. The inputs include preprocessing drugs and proteins as molecular graphs and protein graphs, respectively, and their known DTIs with masked interactions. With the constructed hierarchical heterogeneous graph, two levels of GNNs (LGNN and HGNN) are sequentially designed to capture hierarchical feature embeddings of drugs and proteins, by propagating information from a molecule in a drug or an amino acid in a protein to other drugs and proteins. The high-level encoder incorporates a SAIF module designed to explicitly integrate representations acquired from both a FE and a GE, facilitating consensus representation learning.

In this section, we introduce a novel deep-learning model named H^2^GnnDTI for predicting DTIs. Figure 1 provides an overview of the proposed H^2^GnnDTI. Starting with the structures of drugs and targets, H^2^GnnDTI first constructs a hierarchical heterogeneous graph. It uses a low-level encoder to extract structural and chemical features, followed by a high-level encoder to capture interactive features. Finally, DTI predictions are made using a decoder that utilizes the latent features extracted from the graph.

### 2.1 Problem statement

The DTIs can be formulated as an undirected heterogeneous DTI graph G=(V,E), where *V* represents the set of nodes that contain two disjoint sets of entities from drugs set D and protein set P; *E* denotes the set of edges representing DTIs. The goal of the DTI prediction is to learn a mapping function τ:E→[0,1] from edges to scores, where τ is a function with learnable parameter of Θ, such that we can determine the probability of drug–target pairs having responses. *G* can be represented by node attributes *X* and an adjacency matrix *A*, where A(d,p)=1 if drug *d* has interaction with protein *p* and A(d,p)=0 otherwise. We denote number of drugs |D|=M and number of proteins |P|=N.

### 2.2 Hierarchical heterogeneous graph construction

In this work, the hierarchical graph is defined as a high-level heterogeneous graph with each node being a low-level graph. Specifically, the nodes in the high-level graph are drugs and proteins, and the edges are the known DTIs. Each drug or protein node is further represented as a low-level graph with nodes being molecules in each drug or amino acids in each protein with their chemical descriptors. The low-level graphs for drugs and proteins contain their specific structural and chemical information, while the high-level graph involves the binding information.

For the low-level drug graph, each drug is represented as a graph from its atomic sequences. Suppose there are *m* atoms in a drug, and each atom is described by $\mu_{1}$ kinds of atomic properties, such as the atomic symbol, the atomic degree, the total number of hydrogen atoms, the implicit valence of the atom, whether the bond of the atom is aromatic, etc. The whole list of the characteristics are listed in Supplementary Table S1. The SMILES (Weininger 1988) is a common method for drugs that uses 1D ASCII strings to describe the 3D chemical structure of drugs. In order to take full advantage of the domain knowledge of bio-entities to initialize the node attributes, we use the RDKit technology (an open-source chemistry software) (Bento *et al.* 2020) to convert a drug into a drug molecular graph $G_{d}=\left(V_{d},X_{d},A_{d}\right)$, where $V_{d}$ is the set of drug atom nodes, $X_{d}\in R^{m\times \mu_{1}}$ is a feature matrix containing the properties of all atoms, and $A_{d}$ is an m×m adjacency matrix represents the chemical bonds between atoms, i.e. the graph’s edges, in the form of an adjacency matrix.

For the low-level protein graph, each protein is represented as a graph from its amino acid residue sequence. Suppose there are *n* residues in a protein, and each residue is described with $\mu_{2}$ kinds of physicochemical properties. A protein graph $G_{p}=\left(V_{p},X_{p},A_{p}\right)$ is constructed to model the relationship between residues, where $V_{p}$ is the set of amino acid residues nodes, $X_{p}\in R^{n\times \mu_{2}}$ is a feature matrix containing the physicochemical properties of all residues. All the properties used are listed in Supplementary Table S2. $A_{p}$ is an n×n adjacency matrix representing the connectivity obtained from the protein sequence. The adjacency matrix $A_{p}\in {\left\{0,1\right\}}^{n\times n}$ in the protein graph and protein contact map are exactly equivalent. Contact maps are obtained with atomic level 3D coordinates of proteins. Protein structure information encompasses the angles and distances between different residue pairs, and contact maps are a result of structure prediction. Generally, if the distance between two atoms of residues in Euclidean space is less than a certain threshold (Wu *et al.* 2020), they are considered in contact with each other. We can use Pconsc4 (an open-source) (Michel *et al.* 2019) to predict contact maps. Next, a protein graph can be obtained by using a threshold of 0.5 to generate the corresponding adjacency matrix. In this graph, residues represent nodes, and the relationships between residues are represented by the adjacency matrix. Each residue node feature is determined by the different functional groups it contains. For a protein sequence with amino acid residues, we use the position-specific scoring matrix as its descriptor introduced by Jones (1999).

For the high-level view, a set of drug graphs and protein graphs can be interconnected within a DTI graph $G_{h}=\left(V_{h},X_{h},A_{h}\right)$, where $V_{h}={\left\{G_{d}\right\}}_{d}\cup {\left\{G_{p}\right\}}_{p}$. The high-level view node attributes is a feature matrix containing the properties of all drug and protein graphs, where *f* is an encoder of graph. The *i*th row of the feature matrix $X_{h}$ represents the representation vector for the *i*th drug graph $G_{d}$ or the protein graph $G_{p}$. The edges in the high-level graph contains compulsory connections between drugs and proteins, optional connections among drugs and those among proteins, and the adjacent matrix $A_{h}$ is an (M+N)×(M+N) matrix $A_{h}=\left(\begin{array}{cc} S_{D} & A\\ A^{T} & S_{P} \end{array}\right),$ where *A* denotes the bipartite DTIs among *M* drugs and *N* proteins, $S_{D}$ represents drug similarities and $S_{P}$ represents protein similarities. In some cases, especially when the number of drugs and targets are small, drug similarities and protein similarities could be involved in the high-level graph to aggregate information sufficiently. In this case, drug or protein similarities $S_{D}$ or $S_{P}$ could be calculated from the common number of DTI neighbors. Specifically, if the number of two nodes’ common DTI neighbor is greater than a threshold θ, the two nodes is connected. A function Ψ could be used to compute the common neighbor of two nodes (Wang *et al.* 2021).

### 2.3 LGNN for learning low-level representations

We use the LGNNs to learn the drug or protein representations. Graph convolutional networks (GCNs) have shown great success for relational data and are suitable for learning graph-structured protein representations. Thus, we propose LGNN based on GCNs. Given the adjacency matrix $A_{d}\in {\left\{0,1\right\}}^{m\times m}$ and the feature matrix $X_{d}$ of an arbitrary drug graph $G_{d}$, LGNN adopt a GCN on the molecular graph to learn atom representations which then are summarized into a drug feature vector through a global mean pooling (GEP).Concretely, the *k*th layer of the GCN encoder is defined as:

(1) $$H^{\left(k\right)}=\mathrm{ReLU}({\tilde{D}}^{-\frac{1}{2}}(A_{d}+I_{m}){\tilde{D}}^{-\frac{1}{2}}H^{\left(k-1\right)}W_{L}^{\left(k\right)}),$$

where $I_{m}\in R^{m\times m}$ is the identity matrix, $\tilde{D}\in R^{m\times m}$ is the diagonal degree matrix with entries $D_{aa}=\Sigma_{b}{\left(A_{d}+I_{m}\right)}_{ab}$, $W_{L}^{\left(k\right)}\in R^{\mu_{1}\times d_{1}}$ is a learnable weight matrix for the GCN layer, with $d_{1}$ representing the characteristic dimension of each drug molecular after the GCN encoder. ReLU denotes the activation function, and $H^{\left(0\right)}=X_{d}$. We then apply the GEP overall molecular graphs, we obtain all drug features that can be compiled into $X_{D}\in R^{|D|\times d_{1}}$. To clarify, we use $x_{i}\in R^{1\times d_{1}}$ to represent the final representation for the *i*th drug graph. Similarly, we can obtain the final representation for the *j*th protein graph.

### 2.4 HGNN for learning high-level DTI network

We use the HGNNs to learn DTI network information. HGNN mainly consists of three parts, i.e. a FE, a GE, and a SAIF module (shown in Fig. 1). Based on the features extracted from low-level view, which accounts for structural and chemical information for drugs and proteins, HGNN aims to further capture the interactive features among them.

Formally, we are given the DTI graph $G_{h}=(V_{h},X_{h},A_{h})$, where $X_{h}\in R^{(M+N)\times d_{1}}$ is defined as the feature matrix whose row vector is a final protein representation from LGNN (i.e. $X_{h}^{\left[i,:\right]}=x_{i},i=1,2,\ldots ,M+N$). HGNN updates the representation of the high-level graph by a FE and a GE simultaneously.

#### 2.4.1 Feature encoder

We first construct a FE with an *L*-layer to encode $X_{h}$ via

(2) $$Z_{FE}^{\left(l\right)}=\Phi (W_{H,F}^{\left(l\right)}Z_{FE}^{\left(l-1\right)}+b^{\left(l\right)}),$$

where $H^{\left(0\right)}$ denotes the final representation $X_{h}$ of the low-level view from LGNN, and the activation function $\Phi (x)=\text{LeakyReLU}(x)=\{\begin{array}{cc} x, & \text{if}\;x\geq 0\\ \alpha x, & \text{if}\;x<0. \end{array}$.

#### 2.4.2 Graph encoder

To better make use of both the adjacency information and the attribute information, we design a GE. In the proposed GE, a layer in the encoder is formulated as:

(3) $$Z_{GE}^{\left(l\right)}=\sigma_{1}(D^{-\frac{1}{2}}(A+I)D^{-\frac{1}{2}}Z_{GE}^{\left(l-1\right)}W_{H,G}^{\left(l\right)}),$$

where $D\in R^{\left(M+N)\times (M+N\right)}$ is the diagonal degree matrix with entries $D_{ii}=\Sigma_{j}{\left(A+I\right)}_{ij}$, $W_{H,G}^{\left(l\right)}$ denotes the learnable parameters of the *l*th encoder layer, and $\sigma_{1}$ is a nonlinear activation function $\mathrm{Tanh}(x)=\frac{e^{x}-e^{-x}}{e^{x}+e^{-x}}$.

#### 2.4.3 SAIF module

Inspired by deep fusion clustering method DFCN (Gong *et al.* 2022), to sufficiently explore the graph structure and node attributes information extracted by the FE and GE, we take the strategy of the SAIF.

The information integration within our fusion module includes four steps. First, we combine the latent embedding of FE ($Z_{FE}\in R^{(M+N)\times d_{2}}$) and GE ($Z_{GE}\in R^{(M+N)\times d_{2}}$) with a linear combination operation:

(4) $$Z_{I}=\alpha Z_{FE}+(1-\alpha )Z_{GE},$$

where $d_{2}$ is the latent embedding dimension, and α is a learnable coefficient which selectively determines the importance of two information sources according to the property of the corresponding dataset. In this work, α is initialized as 0.5 and then tuned automatically with a gradient decent method.

Then, we process the combined information with a graph convolution-like operation (i.e. message passing operation). With this operation, we enhance the initial fused embedding $Z_{I}\in R^{(M+N)\times d_{2}}$ by considering the local structure within data:

(5) $$Z_{L}=D^{-\frac{1}{2}}(A+I)D^{-\frac{1}{2}}Z_{I},$$

where $Z_{L}\in R^{(M+N)\times d_{2}}$ denotes the local structure enhanced $Z_{I}$.

We further introduce a self-correlated learning mechanism to exploit the nonlocal relationship in the preliminary information fusion space among samples. Specifically, we first calculate the normalized self-correlation matrix $S\in R^{\left(M+N)\times (M+N\right)}$ through:

(6) $$S_{ij}=\frac{e^{{\left(Z_{L}Z_{L}^{T}\right)}_{ij}}}{\Sigma_{k=1}^{M+N}e^{{\left(Z_{L}Z_{L}^{T}\right)}_{ik}}}.$$

Taking *S* as coefficients, we recombine $Z_{L}$ by considering the global correlation among samples, and adopt a skip connection to encourage information to pass smoothly within the fusion mechanism:

(7) $$\tilde{Z}=\beta SZ_{L}+Z_{L},$$

where β is a scale parameter. Technically, our fusion mechanism considers the sample correlation in both the perspective of the local and global level. Thus, it has a potential benefit on finely fusing and refining the information from both FE and GE for learning consensus latent representations.

### 2.5 Prediction and loss function

The prediction block consists of the GE decoder, which can reconstruct the adjacency matrix. Inspired by the idea of graph autoencoder, we design a graph decoder that is symmetric to the GE mentioned above:

(8) $${\tilde{Z}}^{\left(h\right)}=\sigma_{1}(D^{-\frac{1}{2}}(A+I)D^{-\frac{1}{2}}{\tilde{Z}}^{\left(h-1\right)}W^{\left(h\right)}),$$

where $W^{\left(h\right)}$ denote the learnable parameters of the *h*th decoder layer, and ${\tilde{Z}}^{\left(0\right)}=\tilde{Z}$. The DTI graph is finally reconstructed by

(9) $$\hat{y}=\sigma_{2}({\tilde{Z}}^{\left(h\right)}{\tilde{Z}}^{(h)⊤})$$

from the final layer ${\tilde{Z}}^{\left(h\right)}$, and $\sigma_{2}$ is the sigmoid activation function $\sigma_{2}(x)=\frac{1}{1+e^{-x}}$.

Let $\hat{y}$ be the likelihood of masked interactions in the training set. As a binary classification task, we use binary cross entropy loss to train the model:

(10) $$\mathrm{loss}=-\mathrm{ylog}(\hat{y})-(1-y)\log(1-\hat{y}),$$

where *y* is the ground truth label for the masked interactions. To infer interactions for new drugs or targets, paired drug and target sequences can be placed in the masked part of the adjacency matrix, forming a large hierarchical heterogeneity graph with the original known data. Then the trained model is used to extract features, and the masked position in the reconstructed adjacency matrix is the predicted result for the new drug or target.

## 3 Experiments

### 3.1 Datasets

We evaluated our H^2^GnnDTI on three real datasets: DrugBank, Davis and KIBA. The DrugBank database (Wishart *et al.* 2006) that was released on 3 January 2020 (version 5.1.5). We removed the drugs which are inorganic compounds, very small molecule compounds or those of which the SMILES string cannot be recognized by RDKit python package (Bento *et al.* 2020). Finally, 6647 drugs, 4294 proteins, and 17 511 positive DTIs are obtained in total. Following the work in Huang *et al.* (2021b) and Wen *et al.* (2017), we randomly sampled positive samples and generated the same number of the negative DTI pair from unknown pairs since no negative DTI pairs are provided. Davis (Davis *et al.* 2011) and KIBA (Tang *et al.* 2014) are two unbalanced benchmark datasets, which provide the binding affinity for every drug–target pair. Following early works (Öztürk *et al.* 2018, Zhao *et al*. 2022), the thresholds 5.0 and 12.1 are set for the Davis and KIBA datasets, respectively, to construct binary classification datasets. The detailed statistics of the three datasets are shown in Table 1.

**Table 1.** Statistics of datasets used in our experiments.

Datasets	DrugBank	Davis	KIBA
Drug	6647	68	2068
Protein	4294	379	225
Interaction	35022	25772	116350
Positive DTIs	17511	7320	22154
Negative DTIs	17511	18452	94196

### 3.2 Comparison methods

We evaluated our H^2^GnnDTI by comparing it with seven state-of-the-art methods as follows.

- **GNN-CPI (** Tsubaki *et al.* 2019 **)**: GNN-CPI uses a GNN to encode drugs and uses 1D-CNNs to encode proteins. The latent vectors are then concatenated into a neural network for compound–protein interaction prediction.
- **GNN-PT (** Wang *et al.* 2020 **)**: GNN-PT utilizes GNN to extract drug features and uses Transformer and CNN for protein representation. To capture long-range interaction information within proteins, attention mechanisms are incorporated into protein representation modules for CPI modeling.
- **DeepEmbedding-DTI (** Chen *et al.* 2021 **)**: DeepEmbedding-DTI utilizes end-to-end representation learning of GNNs with attention mechanism and bidirectional long short-term memory (BiLSTM) to predict DTI.
- **DeepConv-DTI (** Lee *et al.* 2019 **)**: DeepConv-DTI captures local residue patterns of targets by convolutions on various lengths of amino acid subsequences. It extracts drug features by using fully connected dense layers on drug fingerprints.
- **TransformerCPI (** Chen *et al.* 2020 **)**: TransformerCPI regards drugs and proteins as two kinds of sequences. The features of target sequences are encoded to the decoder. At the same time, the decoder predicts DTIs based on the output of the encoder and the high-levelological features of drugs extracted by GNN.
- **GraphDTA (** Nguyen *et al.* 2019 **)**: Compared with DeepDTA, GraphDTA also use the paired drug and target input to calculate its corresponding affinity value, the difference is that the drug is converted into a molecular graph, and then a GNN is used on the graph to learn the representation.
- **MolTrans (** Huang *et al.* 2021a **)**: MolTrans constructs the sub-structural pattern mining algorithm. Then, the interaction map is built from drug sequences and target sequences. Finally, the CNN layer is applied on the interaction map to extract higher-order interactions for predicting DTIs.
- **MCL-DTI (** Qian *et al.* 2023 **)**: MCL-DTI combines molecular graph and chemical text data to create comprehensive drug representations. This model adopts a bidirectional multi-head cross attention mechanism and has two independent drug and target decoders.

We follow the same hyper-parameter setting described in the original work for these comparison methods.

### 3.3 Experimental settings

To evaluate the performance of our method, we randomly divided the dataset into training and testing sets with 4:1 ratio. We set up three tasks for DTI prediction, which are commonly encountered in real-world applications. In each setting, different segmentation strategies are used.

- New-drug (S1). Only drug segmentation is used, with interactions between the training drugs and all targets to train the model, which then predicts interactions for test drugs.
- New-target (S2). Only the target segmentation is applied, with interactions between the training targets and all drugs to train the model, which then predicts interactions for test targets.
- New-drug&New-target (S3). Both the drug and target segmentation are used, with the interactions between the training drugs and training targets to train the model, which is then used to predict interactions between the test targets and test drugs.

The H^2^GnnDTI model is trained using the Adam optimizer with a learning rate of 1e−4, and the total epochs is 200. Davis and KIBA are unbalanced benchmark datasets, due to the fact that the positive correlation comes from real interactive datasets, the initial data of DTI is very sparse, we randomly selected negative samples from unrecognized drug target pairs. In the experiment, we set the ratio of positive and negative samples to 1:1. The linear combination parameter α is set to 0.5, and the skip connection parameter β is set to 0.7. For the three datasets, the graph embedding dimension ($d_{1}$) of the drug and protein graph is 80, the embedding dimension ($d_{2}$) of the GE is 20, and the number of layers (*L*) of the GE is 3. Another hyperparameter is the common neighbor threshold, θ, for constructing drug similarity graph and protein similarity graph, optionally. Due to the large size of Drugbank dataset, the formed adjacency matrix is too large, we only take known DTIs as the high-level edges to save computational time. For the Davis and KIBA dataset, θ is 5 and 3, respectively.

We adopt accuracy (ACC), area under the ROC curve (AUC), and area under the precision-recall curve (AUPR) and F1 as the metrics to measure the performance of models.

### 3.4 Performance evaluation under *de novo* setting

**Figure 2.**
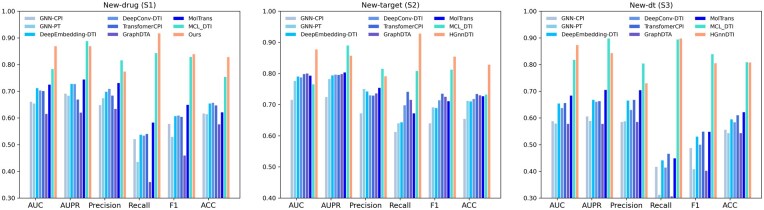
DTI prediction results with other methods under three settings on DrugBank dataset.

Figure 2 depicts the AUC, AUPR, Precison, Recall, F1, and Acc on DrugBank dataset for the three settings, respectively. H^2^GnnDTI consistently achieves the highest or second highest values for these metrics among all evaluated methods, showing its strong performance for identifying potential DTIs. This highlights its superior predictive accuracy and robustness compared to other state-of-the-art methods, particularly in challenging scenarios such as new-drug and new-target settings. While the precision by H^2^GnnDTI tends to be the second best one, the highest Recall highlights its ability to accurately identify true positive DTIs, minimizing the risk of missing potential interactions and thus ensuring comprehensive coverage in the prediction process.

Table 2 reports the results for the Davis dataset. As shown in this table, H^2^GnnDTI achieved superior performance over the state-of-the-art baselines on the Davis dataset, with capability to handle realistic situation on drug discovery. Especially, for the challenging New-target setting S2 and New-drug-New target setting S3, where the performance of comparison method tends to decrease dramatically compared to setting S1, our method could perform well and improve the AUC values (and other values) over other methods. The reason is that our model utilizes hierarchical DTI information and fusion mechanism to dynamically adjust the different features combinations of drugs and proteins, compared to the baselines in which the extracted features of drugs and proteins are often fixed.

**Table 2.** The four metrics obtained by H^2^GnnDTI and several compared methods in the Davis dataset under three settings, including S1 (New-drug), S2 (New-target), and S3 (New-drug&New-target).^a^

Settings metrics	New-drug				New-target				New-dt			
	AUC	AUPR	F1	ACC	AUC	AUPR	F1	ACC	AUC	AUPR	F1	ACC
GNN-CPI	0.812	0.647	0.555	0.785	0.592	0.406	0.389	0.634	0.548	0.378	0.330	0.624
GNN-PT	0.787	0.601	0.536	0.771	0.705	0.522	0.451	0.734	0.612	0.408	0.329	0.656
DeepEmbedding-DTI	0.830	0.691	0.614	0.796	0.705	0.517	0.456	0.727	0.642	0.426	0.362	0.716
DeepConv-DTI	0.845	0.692	0.631	0.800	0.673	0.455	0.460	0.701	0.640	0.420	0.381	0.640
TransfomerCPI	0.842	0.702	0.649	0.807	0.685	0.479	0.477	0.697	0.639	0.412	0.390	0.672
GraphDTA	0.802	0.641	0.514	0.789	0.690	0.451	0.342	0.695	0.605	0.360	0.206	0.675
MolTrans	0.791	0.621	0.696	0.774	0.661	0.452	0.406	0.711	0.642	0.419	0.391	0.648
MCL-DTI	0.866	0.847	0.768	0.792	0.852	0.846	0.780	**0.780**	0.776	0.725	0.648	0.697
Ours	**0.883**	**0.887**	**0.803**	**0.811**	**0.863**	**0.879**	**0.790**	0.776	**0.872**	**0.884**	**0.808**	**0.778**

a The best result is marked in bold and the second one is marked with underline.

Table 3 reports the results for the KIBA dataset. For this dataset, our model outperforms other baseline methods in terms of AUPR and F1 scores significantly for the new-drug and new-target settings, with improvement up to 0.06 for AUPR and 0.09 for F1. However, H^2^GnnDTI tends to produce less competitive AUC and ACC values, possibly due to the large difference in the number of drugs and proteins in the KIBA data, resulting in a highly imbalanced segmentation of the training and testing sets.

**Table 3.** The four metrics obtained by H^2^GnnDTI and several compared methods in the KIBA dataset under three settings, including S1 (New-drug), S2 (New-target), and S3 (New-drug&New-target).^a^

Settings metrics	New-drug				New-target				New-dt			
	AUC	AUPR	F1	ACC	AUC	AUPR	F1	ACC	AUC	AUPR	F1	ACC
GNN-CPI	0.770	0.503	0.491	0.801	0.792	0.512	0.487	0.820	0.686	0.357	0.398	0.732
GNN-PT	0.732	0.474	0.444	0.804	0.814	0.522	0.489	0.828	0.651	0.312	0.336	0.755
DeepEmbedding-DTI	0.809	0.586	0.505	**0.846**	0.792	0.507	0.484	0.808	0.706	0.379	0.369	0.780
DeepConv-DTI	0.788	0.547	0.535	0.833	0.812	0.565	0.611	0.837	0.732	0.438	0.460	0.806
TransfomerCPI	0.799	0.555	0.528	0.830	0.822	0.581	0.522	0.833	0.717	0.416	0.404	0.795
GraphDTA	0.754	0.495	0.505	0.836	0.838	0.619	0.556	**0.849**	0.678	0.374	0.270	0.821
MolTrans	0.781	0.525	0.543	0.836	0.817	0.573	0.604	0.847	0.726	0.428	0.460	0.807
MCL-DTI	**0.841**	0.770	0.702	0.781	**0.841**	0.767	0.689	0.787	**0.912**	**0.862**	**0.795**	**0.839**
Ours	0.798	**0.812**	**0.743**	0.699	**0.841**	**0.829**	**0.774**	0.751	0.809	0.836	0.792	0.728

a The best result is marked in bold and the second one is marked with underline.

### 3.5 Ablation study

To investigate the individual role of each component in the proposed model, we conducted a series of ablation experiments by testing several variants of H^2^GnnDTI:

- H^2^GnnDTI-L removes the LGNN module and simply uses the original drug/protein sequences. It starts with label encoding to transform each SMILES character and amino acid to the corresponding integer labels, in order to process sequences in batches, the maximum length of the drug is set to 100, parts with a length exceeding 100 will be truncated, strings with a length <100 are padded with 0.The maximum length of an amino acid sequence is 1000.
- H^2^GnnDTI-F only retains the GE in the HGNN, and removes the SAIF fusion mechanism.
- H^2^GnnDTI-W simply replaces the learnable weight α in Equation (4) by 0.5, i.e. uses the average of the FE and GE as $Z_{I}$ in the HGNN.

The performance comparison of H^2^GnnDTI with its three ablation variants on the Davis dataset is shown in Table 4. The experimental results indicate that our proposed model achieves the best performance compared to these variants, demonstrating the individual contributions of our model’s two-level hierarchical view, as well as the advantages of fusion strategies. Among these variant models, the performance degradation of the H^2^GnnDTI-F variant is the most significant, proving that the fusion mechanism is the most critical component of our proposed model. For the S2 task, H^2^GnnDTI-W performs similarly to H^2^GnnDTI, suggesting that the learnable weight α for the GE and FE in Equation (4) may have a marginal contribution in certain tasks, and that simply averaging could achieve similar results.

**Table 4.** Ablation study in the Davis dataset under three settings, including S1 (New-drug), S2 (New-target), and S3 (New-drug&New-target).^a^

Settings metrics	New-drug				New-target				New-dt			
	AUC	AUPR	F1	ACC	AUC	AUPR	F1	ACC	AUC	AUPR	F1	ACC
H^2^GnnDTI	**0.883**	**0.887**	**0.803**	**0.811**	0.863	**0.879**	**0.790**	0.776	**0.872**	**0.884**	**0.808**	**0.778**
H^2^GnnDTI-L	0.879	0.884	0.798	0.797	0.857	0.874	0.787	0.775	0.867	0.881	0.803	0.767
H^2^GnnDTI-F	0.868	0.869	0.794	0.791	0.859	0.871	0.783	0.761	0.852	0.864	0.792	0.750
H^2^GnnDTI-W	0.871	0.869	0.795	0.793	**0.865**	0.873	0.786	**0.781**	0.855	0.864	0.799	0.756

a The best result is marked in bold and the second one is marked with underline.

### 3.6 Parameter sensitivity analysis

In our work, one hyperparameter is the common neighbor threshold θ, which is used to construct the drug–drug edges and target-target edges in the high-level graph. For example, θ=1 means that two nodes are connected if they have at least one common neighbor.

**Figure 3.**
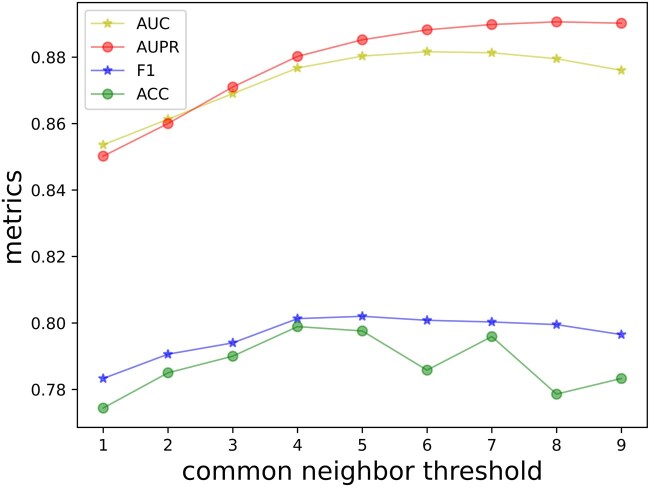
Performance evaluation on using different common neighbor threshold for DTI prediction under new-drug setting S1 on the Davis dataset.

We checked the influence of common neighbors under New-drug setting S1 on the Davis dataset. The results of different settings were shown in Fig. 3. From the figure, we could see that a very small θ produces lower metrics. Since in DTI network, many different drugs may interact with the same protein as target, a very small θ may cause most drugs to be connected, which destroy the similarity information (Wang *et al.* 2021). As the θ increases, the DTI graph involves more useful similarity edges, which leads to the accuracy increase, and when θ is too big, very few connections between drugs or targets could be detected, which leads to the accuracy decrease. For the Davis and KIBA dataset, θ is 5 and 3 respectively.

In addition, the graph embedding dimension ($d_{1}$) of the drug and protein graph, the embedding dimension ($d_{2}$) of the GE, and the number of layers (*L*) of the GE will affect the quality of the node embedding representation. These results are available in Supplementary Material.

## 4 Conclusion

In this work, we introduce H^2^GnnDTI, a hierarchical graph deep learning approach for predict DTIs, which could simultaneously learn low-level structural and chemical features by low-level GNNs and high-level interactive features by high-level a GNN.

H^2^GnnDTI works like biologists in a hierarchical manner as it contains the two-level view of drug and protein. On one hand, H^2^GnnDTI applies the low-level view when dealing with a drug/protein, which is represented by a drug/protein graph. On the other hand, from the high-level view, protein graphs and their interactions are considered nodes and edges of the DTI graph, respectively. Correspondingly, two GNNs are respectively used to learn from drug/protein graphs in the LGNNs and learn from a DTI graph in the HGNNs. Consequently, a set of graphs are interconnected by edges in a hierarchical graph, to better present the data representation. Moreover, the component SAIF module in the high-level GNN leverages both graph structure and node attributes via a suitable fusion mechanism. In this way, more consensus and discriminative information from both sides is encoded to construct the robust features and improve the generalization capability of the proposed method. Experiments on three benchmark datasets show that H^2^GnnDTI consistently outperforms state-of-the-art baseline methods. In the future, we plan to further improve our method to adapt it to more new drug prediction scenarios.

## Supplementary Material

btaf117_Supplementary_Data

## Data Availability

The preprocessed data used in this work is available at https://github.com/LiminLi-xjtu/H2GnnDTI/blob/master/dataset.rar.
