# Supplementary material for: H2GnnDTI: hierarchical heterogeneous graph neural networks for drug–target interaction prediction
Source: Bioinformatics. 2025 Mar 17;41(4):btaf117. doi: 10.1093/bioinformatics/btaf117 (PMC11954568; doi:10.1093/bioinformatics/btaf117)
Supplement: btaf117_Supplementary_Data [file btaf117_supplementary_data.pdf]

Supplementary Materials for "H^2GnnDTI: Hierarchical heterogeneous graph neural networks for drug target interaction prediction"

1 The details of all properties

Table 1 lists the μ_1 kinds of atom features of molecular graph. Table 2 lists the μ_2 kinds of amino acid residue features of protein graph.

Table 1: atom properties

Feature	Dimension
One-hot encoding of the atom element	44
One-hot encoding of the degree of the atom in the molecule, which is the number of directly-bonded neighbors(atoms)	11
One-hot encoding of the total number of H bound to the atom	11
One-hot encoding of the number of implicit H bound to the atom	11
Whether the atom is aromatic	1

Table 2: residue properties

Feature	Dimension
One-hot encoding of the atom element	21
Position-specics coring matrix(PSSM)	21
Whether the residue is aliphatic	1
Whether the residue is aromatic	1
Whether the residue is polar neutral	1
Whether the residue is acidic charged	1
Whether the residue is basic charged	1
Residue weight	1
The negative of the logarithm of the dissociation constant for the–COOH group	1
The negative of the logarithm of the dissociation constant for the–NH3 group	1
The negative of the logarithm of the dissociation constant for anyother group in the molecule	1
The pH at the isoelectric point	1
Hydrophobicity of residue(pH$\frac{1}{4}$2)	1
Hydrophobicity of residue(pH$\frac{1}{4}$7)	1

2 Parameter Sensitivity Analysis

In this model, the graph embedding dimension (d_1) of the drug and protein graph, the embedding dimension (d_2) of the GE, and the number of layers (L) of the GE will affect the quality of the node embedding representation. We conduct performance evaluation using different parameters under setting S1 on DrugBank dataset.

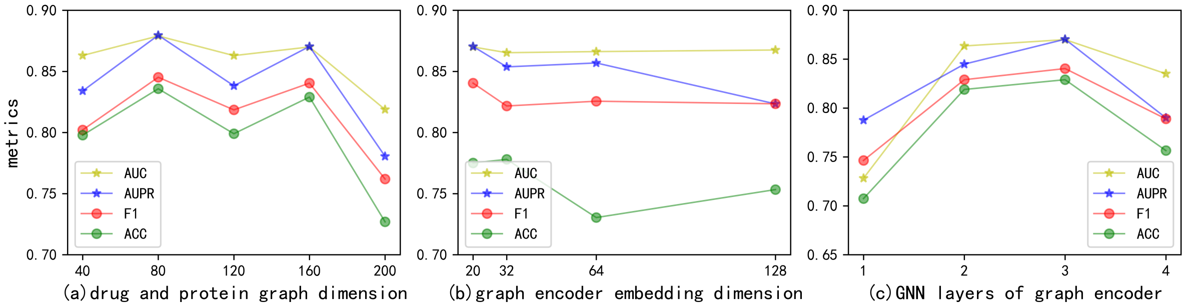

Figure 1: Performance evaluation on using different parameters for DTI prediction under New-drug setting S1 on DrugBank dataset.

2.1 The effect of the node embedding dimension

From Fig.1(a), as the embedding graph dimension of the drug and protein increases, AUC, AUPR, F1, and ACC first increase and then decrease. This is because when d_1 is small, the embeddings learned by drugs and targets through GCN do not fully extract relevant features, resulting in insufficient fitting; When d_1 is too large, the extracted node embeddings may contain noise, causing the model to overfit. In our experiments, we set $d_1 = 80$ for save computational time.

From Fig.1(b), it can be seen that as the embedding dimension of the graph encoder (GE) increases, the ACC first increases and then decreases, but it is increasing slowly. When d_2 is too large, the extracted node embeddings may contain noise, which can also affect prediction performance. AUC, AUPR, and F1 first decrease and then stabilize, which also indicates that our model has good stability with different dimensions. Considering the time cost of training, using a large embedding dimension is not beneficial. Therefore, we set d_2 to 20 in all experiments.

2.2 The effect of the number of GE GNN layers

We also investigated the impact of the number of GNN layers of GE on the Davis dataset. From Fig.1(c), it can be seen that as the number of layers increases, AUC, AUPR, F1, and ACC all increase first and then decrease. When the graph convolution is small, it cannot fully bring the distance between adjacent nodes closer. However, when L is greater than 3, all indicators are decreasing, indicating that excessive smoothing has already occurred. In addition, as the number of layers increases, the training time gradually increases. Therefore, we set the number of graph convolutional layers in GE to 3 in all experiments.
